# 4-Cyano-1-(4-nitro­benz­yl)pyridinium bis­(2-thioxo-1,3-dithiole-4,5-dithiol­ato-κ^2^ *S* ^4^,*S* ^5^)nickelate(III)

**DOI:** 10.1107/S1600536810037426

**Published:** 2010-09-30

**Authors:** Kai-Hui Li, Qing-Duo Lei, Chong-Zhen Mei

**Affiliations:** a Institute of Environmental and Municipal Engineering, North China University of Water Conservancy and Electric Power, Zhengzhou 450011, People’s Republic of China

## Abstract

In the title salt, (C_13_H_10_N_3_O_2_)[Ni(C_3_S_5_)_2_], the Ni^III^ cation is *S*,*S*′-chelated by two 2-thioxo-1,3-dithiole-4,5-dithiol­ate anions in a distorted square-planar geometry. The complex anion is approximately planar with a maximum deviation of 0.097 (1) Å. In the 1-(4-nitro­benz­yl)-4-cyano­pyridinium cation, the pyridine ring is twisted at a dihedral angle of 73.84 (16)° with respect to the benzene ring. π-π stacking is observed between nearly parallel [dihedral angle = 4.71 (7)°] dithiole and benzene rings, the centroid–centroid distance being 3.791 (2) Å.

## Related literature

For background to and applications of dithiol­ate metal complexes, see: Akutagawa & Nakamura (2000 ▶); Cassoux (1999 ▶). For the structure of a complex with a 2-thioxo-1,3-dithiole-4,5-dithiol­ate ligand, see: Zang *et al.* (2006 ▶). For weak inter­molecular inter­actions, see: Egli & Sarkhel (2007 ▶); Tian *et al.* (2007 ▶); Cundari *et al.* (2010 ▶).
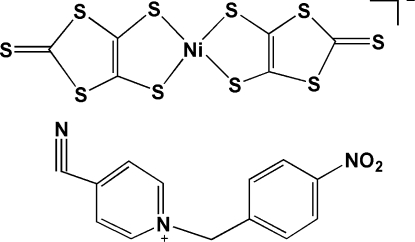


## Experimental

### 

#### Crystal data

(C_13_H_10_N_3_O_2_)[Ni(C_3_S_5_)_2_]
*M* *_r_* = 691.61
Monoclinic, 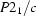

*a* = 8.4896 (17) Å
*b* = 25.789 (5) Å
*c* = 12.043 (3) Å
β = 106.181 (3)°
*V* = 2532.3 (9) Å^3^
*Z* = 4
Mo *K*α radiation
μ = 1.62 mm^−1^
*T* = 296 K
0.20 × 0.17 × 0.15 mm

#### Data collection

Bruker SMART APEXII CCD area detector diffractometer
Absorption correction: multi-scan (*SADABS*; Bruker, 2005 ▶) *T*_min_ = 0.738, *T*_max_ = 0.794
12415 measured reflections
4423 independent reflections
3343 reflections with *I* > 2σ(*I*)
*R*_int_ = 0.043

#### Refinement

*R*[*F* ^2^ > 2σ(*F* ^2^)] = 0.040
*wR*(*F* ^2^) = 0.090
*S* = 1.02
4423 reflections
316 parameters
H-atom parameters constrained
Δρ_max_ = 0.57 e Å^−3^
Δρ_min_ = −0.26 e Å^−3^

### 

Data collection: *APEX2* (Bruker, 2005 ▶); cell refinement: *SAINT* (Bruker, 2005 ▶); data reduction: *SAINT*; program(s) used to solve structure: *SHELXS97* (Sheldrick, 2008 ▶); program(s) used to refine structure: *SHELXL97* (Sheldrick, 2008 ▶); molecular graphics: *SHELXTL* (Sheldrick, 2008 ▶); software used to prepare material for publication: *SHELXTL*.

## Supplementary Material

Crystal structure: contains datablocks I, global. DOI: 10.1107/S1600536810037426/xu5018sup1.cif

Structure factors: contains datablocks I. DOI: 10.1107/S1600536810037426/xu5018Isup2.hkl

Additional supplementary materials:  crystallographic information; 3D view; checkCIF report

## Figures and Tables

**Fig. 1.**
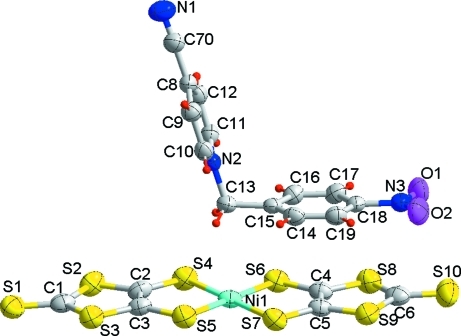
The cation and anion in (I), showing the atom-labelling scheme, with displacement ellipsoids drawn at the 50% probability level.

**Fig. 2.**
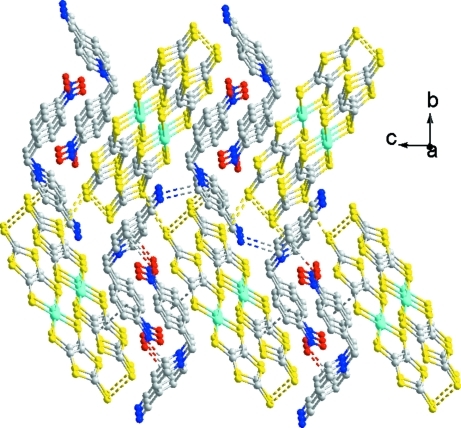
Three-dimensional supramolecular structure of (I). Hydrogen atoms have been omitted for clarity. Dashed lines indicate weak S···S, CN···π, lp···π, donor-acceptor and π···π interactions.

## supplementary crystallographic information

### Comment

Bis-dithiolate metal ion-pair complexes have been actively studied for a long time as a wide range of conducting and magneticmaterials as well as nonlinear opticalmaterials (Cassoux, 1999). 2-Thioxo-1,3-dithiole-4,5-dithiolate metal complex also is excellent building block employed for the construction of molecular magnetic materials (Zang *et al.*, 2006) apart from its well known electric conductivity molecular conductors (Akutagawa & Nakamura, 2000). We report herein the synthesis and crystal structure of the new ion-pair complex.

The title compound (I) comprises [Ni^III^(dmit)_2_]^-^ anions (where dmit = 2-thioxo-1,3-dithiole-4,5-dithiolate) and 1-(4-nitrobenzyl)-4-cyanopyridinium cations (Figure 1). The Ni^III^ ion adopts a square-planar geometry coordinated by four S atoms of two dmit ligands, with Ni—S bond lengths ranging from 2.157 to 2.166 Å. Two [Ni^III^(dmit)_2_]^-^ anions form pairs across centres of inversion, with their least squares planes parallel and Ni1···Ni1^i^= 3.668 Å, S1···S10^i^= 3.636 Å, [symmetry code: (i) -*x*, 1 - *y*, 1 - *z*]. Neighbouring anion pairs are nearly vertical or parallel arrangements so that S···S interactions in this region include S5···S7^ii^ = 3.606, S5···S9^ii^ = 3.473, S7···S5^ii ^= 3.506, S7···S7^ii ^= 3.471 and S10^i^···S2^iii^ = 3.496 Å [symmetry codes: (ii) 1 - *x*, 1 - *y*, 1 - *z*; (iii) *x*, 1.5 - *y*, 1/2 + *z*]. The adjacent [NO_2_CNbzpy]^+ ^cations adopting edge-to-face inversion arrangements are associated together through lone pair-aromatic interactions (lp···π: O2-centroid distance 2.953 Å) (Egli & Sarkhel, 2007) between the oxygen atom of nitro group and the pyridine ring from neighboring cation and CN···π interactions (C10···N1^iii^ distance 3.010 Å) (Tian  *et al.*, 2007) between the CN group at the end of the cations and the pyridine ring of the adjacent cation. In addition, there is π···π interaction (centroid-centroid distance 3.625 Å) between phenyl ring of [NO_2_CNbzpy]^+ ^cation and the ethylene group (C4═C5) of [Ni^III^(dmit)_2_]^-^ anion and donor-acceptor interaction between anion and cation (S1···C11^ iii^ distance 3.234 Å [symmetry codes (iii) *x*, 1.5 - *y*, 1/2 + *z*]) (Cundari *et al.*, 2010). The weak S···S, CN···π, lp···π, donor-acceptor and π···π interactions lead a three-dimensional supramolecular structure (Figure 2).

### Experimental

4,5-Bis(thiobenzoyl)-1,3-dithiole-2-thione (812 mg, 2.0 mmol) was suspended in dry methanol (20 ml) and sodium (92 mg, 4.0 mmol) was added under a nitrogen atmosphere at room temperature to give a bright-red solution. NiCl_2_.6H_2_O (238 mg, 1 mmol) was then added, followed successively by I_2_ (127 mg, 0.5 mmol) and a solution of 1-(4-nitrobenzyl)-4-cyanopyridinium chloride (276 mg, 1 mmol) in methanol at an interval of approximately 20 min. The solution was stirred for a further 30 min and the resulting solid collected by filtration. Single crystals of the title complound were obtained by evaporation of a dilute acetone solution over 2 weeks at room temperature.

### Refinement

H atoms were positioned geometrically and refined using a riding model, with C—H = 0.93 Å, *U*_iso_(H) = 1.2U_eq_(C) for aromatic H, and C—H = 0.97 Å, *U*_iso_(H) = 1.2U_eq_(C) for CH_2_.

### Crystal data

**Table:**

(C_13_H_10_N_3_O_2_)[Ni(C_3_S_5_)_2_]	*F*(000) = 1396
*M**_r_* = 691.61	*D*_x_ = 1.814 Mg m^−^^3^
Monoclinic, *P*2_1_/*c*	Mo *K*α radiation, λ = 0.71073 Å
Hall symbol: -P 2ybc	Cell parameters from 947 reflections
*a* = 8.4896 (17) Å	θ = 2.4–24.3°
*b* = 25.789 (5) Å	µ = 1.62 mm^−^^1^
*c* = 12.043 (3) Å	*T* = 296 K
β = 106.181 (3)°	Block, black
*V* = 2532.3 (9) Å^3^	0.20 × 0.17 × 0.15 mm
*Z* = 4	

### Data collection

**Table:**

Bruker SMART APEXII CCD area detector diffractometer	4423 independent reflections
Radiation source: fine-focus sealed tube	3343 reflections with *I* > 2σ(*I*)
graphite	*R*_int_ = 0.043
φ and ω scans	θ_max_ = 25.0°, θ_min_ = 1.9°
Absorption correction: multi-scan (*SADABS*; Bruker, 2005)	*h* = −9→10
*T*_min_ = 0.738, *T*_max_ = 0.794	*k* = −30→21
12415 measured reflections	*l* = −14→14

### Refinement

**Table:**

Refinement on *F*^2^	Primary atom site location: structure-invariant direct methods
Least-squares matrix: full	Secondary atom site location: difference Fourier map
*R*[*F*^2^ > 2σ(*F*^2^)] = 0.040	Hydrogen site location: inferred from neighbouring sites
*wR*(*F*^2^) = 0.090	H-atom parameters constrained
*S* = 1.02	*w* = 1/[σ^2^(*F*_o_^2^) + (0.0387*P*)^2^ + 0.150*P*] where *P* = (*F*_o_^2^ + 2*F*_c_^2^)/3
4423 reflections	(Δ/σ)_max_ = 0.001
316 parameters	Δρ_max_ = 0.57 e Å^−^^3^
0 restraints	Δρ_min_ = −0.26 e Å^−^^3^

### Special details

**Table:**

Geometry. All e.s.d.'s (except the e.s.d. in the dihedral angle between two l.s. planes) are estimated using the full covariance matrix. The cell e.s.d.'s are taken into account individually in the estimation of e.s.d.'s in distances, angles and torsion angles; correlations between e.s.d.'s in cell parameters are only used when they are defined by crystal symmetry. An approximate (isotropic) treatment of cell e.s.d.'s is used for estimating e.s.d.'s involving l.s. planes.
Refinement. Refinement of *F*^2^ against ALL reflections. The weighted *R*-factor *wR* and goodness of fit *S* are based on *F*^2^, conventional *R*-factors *R* are based on *F*, with *F* set to zero for negative *F*^2^. The threshold expression of *F*^2^ > σ(*F*^2^) is used only for calculating *R*-factors(gt) *etc*. and is not relevant to the choice of reflections for refinement. *R*-factors based on *F*^2^ are statistically about twice as large as those based on *F*, and *R*- factors based on ALL data will be even larger.

### Fractional atomic coordinates and isotropic or equivalent isotropic displacement parameters (Å^2^)

**Table:**

	*x*	*y*	*z*	*U*_iso_*/*U*_eq_	
Ni1	0.12504 (5)	0.535201 (17)	0.42287 (3)	0.04673 (14)	
O1	0.2608 (4)	0.40743 (10)	−0.0690 (2)	0.0797 (9)	
O2	0.5120 (4)	0.39512 (10)	0.0279 (2)	0.0754 (8)	
N1	0.5275 (4)	0.80533 (13)	−0.0531 (3)	0.0746 (10)	
N2	0.4084 (3)	0.64870 (9)	0.1936 (2)	0.0424 (6)	
N3	0.3834 (4)	0.41930 (11)	0.0065 (2)	0.0552 (8)	
S1	0.08507 (13)	0.76215 (4)	0.75005 (9)	0.0690 (3)	
S2	−0.05329 (12)	0.69214 (4)	0.55265 (9)	0.0660 (3)	
S3	0.27096 (11)	0.67162 (4)	0.69657 (8)	0.0602 (3)	
S4	−0.05363 (11)	0.59682 (4)	0.40526 (8)	0.0620 (3)	
S5	0.29798 (11)	0.57297 (4)	0.56561 (8)	0.0578 (3)	
S6	−0.04453 (10)	0.49687 (4)	0.27893 (8)	0.0581 (3)	
S7	0.30541 (10)	0.47438 (4)	0.44187 (8)	0.0559 (3)	
S8	−0.01230 (12)	0.39818 (4)	0.15167 (8)	0.0661 (3)	
S9	0.31466 (11)	0.37927 (4)	0.29563 (8)	0.0596 (3)	
S10	0.16951 (15)	0.30728 (4)	0.10160 (11)	0.0842 (4)	
C1	0.0997 (4)	0.71136 (13)	0.6712 (3)	0.0553 (9)	
C2	0.0396 (4)	0.63686 (13)	0.5171 (3)	0.0514 (9)	
C3	0.1933 (4)	0.62697 (13)	0.5861 (3)	0.0499 (9)	
C4	0.0626 (4)	0.44299 (13)	0.2605 (3)	0.0510 (9)	
C5	0.2162 (4)	0.43352 (13)	0.3297 (3)	0.0487 (8)	
C6	0.1579 (4)	0.35837 (14)	0.1780 (3)	0.0602 (10)	
C8	0.4722 (4)	0.72766 (12)	0.0638 (3)	0.0442 (8)	
C9	0.5968 (4)	0.70593 (12)	0.1495 (3)	0.0480 (8)	
H9	0.7035	0.7184	0.1643	0.058*	
C10	0.5612 (4)	0.66582 (12)	0.2124 (3)	0.0472 (8)	
H10	0.6451	0.6503	0.2693	0.057*	
C11	0.2853 (4)	0.66928 (12)	0.1100 (3)	0.0458 (8)	
H11	0.1793	0.6565	0.0976	0.055*	
C12	0.3145 (4)	0.70877 (12)	0.0432 (3)	0.0484 (8)	
H12	0.2295	0.7228	−0.0153	0.058*	
C13	0.3706 (4)	0.60448 (12)	0.2623 (3)	0.0511 (9)	
H13A	0.2637	0.6097	0.2749	0.061*	
H13B	0.4516	0.6031	0.3372	0.061*	
C14	0.5122 (4)	0.52460 (13)	0.2208 (3)	0.0485 (9)	
H14	0.6052	0.5348	0.2781	0.058*	
C15	0.3716 (4)	0.55425 (11)	0.1992 (2)	0.0404 (7)	
C16	0.2328 (4)	0.53870 (12)	0.1150 (3)	0.0476 (8)	
H16	0.1371	0.5581	0.1013	0.057*	
C17	0.2358 (4)	0.49449 (12)	0.0513 (3)	0.0474 (8)	
H17	0.1435	0.4841	−0.0064	0.057*	
C18	0.3785 (4)	0.46620 (12)	0.0752 (2)	0.0415 (8)	
C19	0.5162 (4)	0.48020 (13)	0.1588 (3)	0.0484 (8)	
H19	0.6109	0.4602	0.1735	0.058*	
C20	0.5035 (4)	0.77070 (14)	−0.0025 (3)	0.0538 (9)	

### Atomic displacement parameters (Å^2^)

**Table:**

	*U*^11^	*U*^22^	*U*^33^	*U*^12^	*U*^13^	*U*^23^
Ni1	0.0354 (2)	0.0605 (3)	0.0427 (3)	0.0008 (2)	0.00814 (19)	0.0116 (2)
O1	0.094 (2)	0.0649 (18)	0.0670 (18)	−0.0028 (15)	0.0001 (16)	−0.0202 (14)
O2	0.083 (2)	0.0650 (18)	0.0817 (19)	0.0176 (16)	0.0288 (16)	−0.0093 (14)
N1	0.084 (2)	0.070 (2)	0.068 (2)	−0.0089 (19)	0.0177 (19)	0.0166 (18)
N2	0.0533 (17)	0.0397 (15)	0.0352 (14)	−0.0012 (13)	0.0137 (13)	−0.0033 (12)
N3	0.075 (2)	0.0465 (19)	0.0471 (18)	−0.0005 (17)	0.0216 (17)	0.0048 (14)
S1	0.0714 (7)	0.0700 (7)	0.0707 (7)	0.0025 (5)	0.0282 (5)	0.0038 (5)
S2	0.0494 (6)	0.0619 (6)	0.0801 (7)	0.0097 (5)	0.0074 (5)	0.0082 (5)
S3	0.0480 (5)	0.0662 (6)	0.0623 (6)	0.0019 (5)	0.0082 (4)	0.0029 (5)
S4	0.0433 (5)	0.0683 (7)	0.0632 (6)	0.0080 (4)	−0.0037 (4)	0.0067 (5)
S5	0.0409 (5)	0.0680 (6)	0.0569 (6)	0.0088 (4)	0.0011 (4)	0.0029 (5)
S6	0.0376 (5)	0.0763 (7)	0.0546 (6)	0.0032 (4)	0.0031 (4)	0.0042 (5)
S7	0.0403 (5)	0.0726 (6)	0.0486 (5)	0.0074 (4)	0.0022 (4)	0.0011 (4)
S8	0.0549 (6)	0.0742 (7)	0.0610 (6)	−0.0100 (5)	0.0026 (5)	−0.0021 (5)
S9	0.0533 (6)	0.0602 (6)	0.0634 (6)	0.0004 (5)	0.0133 (5)	0.0046 (5)
S10	0.0937 (9)	0.0676 (7)	0.0933 (8)	−0.0168 (6)	0.0295 (7)	−0.0164 (6)
C1	0.051 (2)	0.058 (2)	0.062 (2)	−0.0038 (17)	0.0230 (18)	0.0135 (18)
C2	0.0419 (19)	0.051 (2)	0.061 (2)	0.0022 (16)	0.0148 (17)	0.0148 (17)
C3	0.046 (2)	0.051 (2)	0.052 (2)	−0.0036 (16)	0.0117 (16)	0.0121 (16)
C4	0.044 (2)	0.062 (2)	0.047 (2)	−0.0052 (17)	0.0128 (16)	0.0072 (17)
C5	0.0421 (19)	0.060 (2)	0.0463 (19)	−0.0017 (16)	0.0159 (16)	0.0101 (16)
C6	0.060 (2)	0.066 (2)	0.056 (2)	−0.0156 (19)	0.0190 (18)	0.0090 (18)
C8	0.057 (2)	0.0373 (19)	0.0412 (19)	−0.0013 (16)	0.0175 (16)	−0.0019 (15)
C9	0.049 (2)	0.049 (2)	0.046 (2)	−0.0029 (16)	0.0124 (16)	0.0001 (16)
C10	0.052 (2)	0.048 (2)	0.0385 (18)	0.0043 (17)	0.0067 (15)	−0.0020 (16)
C11	0.048 (2)	0.040 (2)	0.051 (2)	−0.0027 (16)	0.0153 (16)	−0.0069 (16)
C12	0.053 (2)	0.043 (2)	0.0460 (19)	0.0058 (16)	0.0087 (16)	−0.0009 (16)
C13	0.071 (2)	0.047 (2)	0.0395 (18)	−0.0047 (18)	0.0208 (17)	0.0023 (16)
C14	0.048 (2)	0.056 (2)	0.0364 (18)	−0.0059 (16)	0.0029 (15)	0.0038 (16)
C15	0.050 (2)	0.0405 (18)	0.0329 (16)	−0.0041 (15)	0.0144 (15)	0.0059 (14)
C16	0.0411 (18)	0.046 (2)	0.055 (2)	0.0015 (15)	0.0122 (16)	0.0055 (17)
C17	0.0404 (19)	0.046 (2)	0.050 (2)	−0.0060 (16)	0.0036 (16)	0.0034 (16)
C18	0.054 (2)	0.0370 (18)	0.0370 (17)	−0.0027 (15)	0.0175 (15)	0.0038 (14)
C19	0.047 (2)	0.050 (2)	0.046 (2)	0.0050 (16)	0.0102 (16)	0.0078 (16)
C20	0.061 (2)	0.052 (2)	0.047 (2)	−0.0031 (18)	0.0120 (18)	0.0024 (18)

### Geometric parameters (Å, °)

**Table:**

Ni1—S5	2.1570 (10)	C2—C3	1.362 (4)
Ni1—S7	2.1591 (10)	C4—C5	1.360 (4)
Ni1—S6	2.1596 (10)	C8—C9	1.375 (4)
Ni1—S4	2.1661 (10)	C8—C12	1.380 (4)
O1—N3	1.215 (3)	C8—C20	1.434 (5)
O2—N3	1.220 (4)	C9—C10	1.365 (4)
N1—C20	1.132 (4)	C9—H9	0.9300
N2—C10	1.328 (4)	C10—H10	0.9300
N2—C11	1.343 (4)	C11—C12	1.362 (4)
N2—C13	1.495 (4)	C11—H11	0.9300
N3—C18	1.473 (4)	C12—H12	0.9300
S1—C1	1.643 (4)	C13—C15	1.503 (4)
S2—C1	1.714 (4)	C13—H13A	0.9700
S2—C2	1.740 (4)	C13—H13B	0.9700
S3—C1	1.735 (4)	C14—C19	1.373 (4)
S3—C3	1.744 (3)	C14—C15	1.379 (4)
S4—C2	1.707 (4)	C14—H14	0.9300
S5—C3	1.707 (4)	C15—C16	1.383 (4)
S6—C4	1.709 (4)	C16—C17	1.378 (4)
S7—C5	1.714 (3)	C16—H16	0.9300
S8—C6	1.728 (4)	C17—C18	1.375 (4)
S8—C4	1.730 (3)	C17—H17	0.9300
S9—C5	1.736 (4)	C18—C19	1.362 (4)
S9—C6	1.738 (4)	C19—H19	0.9300
S10—C6	1.626 (4)		
			
S5—Ni1—S7	86.47 (4)	C9—C8—C20	120.7 (3)
S5—Ni1—S6	178.98 (4)	C12—C8—C20	119.6 (3)
S7—Ni1—S6	92.69 (4)	C10—C9—C8	118.9 (3)
S5—Ni1—S4	92.83 (4)	C10—C9—H9	120.6
S7—Ni1—S4	179.28 (4)	C8—C9—H9	120.6
S6—Ni1—S4	88.01 (4)	N2—C10—C9	120.9 (3)
C10—N2—C11	121.1 (3)	N2—C10—H10	119.5
C10—N2—C13	120.6 (3)	C9—C10—H10	119.5
C11—N2—C13	118.3 (3)	N2—C11—C12	120.4 (3)
O1—N3—O2	124.0 (3)	N2—C11—H11	119.8
O1—N3—C18	118.4 (3)	C12—C11—H11	119.8
O2—N3—C18	117.7 (3)	C11—C12—C8	119.0 (3)
C1—S2—C2	98.72 (17)	C11—C12—H12	120.5
C1—S3—C3	97.80 (16)	C8—C12—H12	120.5
C2—S4—Ni1	102.02 (12)	N2—C13—C15	110.2 (2)
C3—S5—Ni1	102.58 (11)	N2—C13—H13A	109.6
C4—S6—Ni1	102.50 (11)	C15—C13—H13A	109.6
C5—S7—Ni1	102.63 (12)	N2—C13—H13B	109.6
C6—S8—C4	98.39 (16)	C15—C13—H13B	109.6
C5—S9—C6	97.64 (17)	H13A—C13—H13B	108.1
S1—C1—S2	123.4 (2)	C19—C14—C15	120.7 (3)
S1—C1—S3	124.3 (2)	C19—C14—H14	119.6
S2—C1—S3	112.3 (2)	C15—C14—H14	119.6
C3—C2—S4	121.6 (3)	C14—C15—C16	119.5 (3)
C3—C2—S2	115.2 (3)	C14—C15—C13	120.6 (3)
S4—C2—S2	123.19 (19)	C16—C15—C13	119.8 (3)
C2—C3—S5	120.9 (3)	C17—C16—C15	120.3 (3)
C2—C3—S3	115.9 (3)	C17—C16—H16	119.9
S5—C3—S3	123.24 (19)	C15—C16—H16	119.9
C5—C4—S6	121.4 (3)	C18—C17—C16	118.3 (3)
C5—C4—S8	115.6 (3)	C18—C17—H17	120.9
S6—C4—S8	123.02 (19)	C16—C17—H17	120.9
C4—C5—S7	120.7 (3)	C19—C18—C17	122.6 (3)
C4—C5—S9	116.3 (3)	C19—C18—N3	118.7 (3)
S7—C5—S9	123.06 (19)	C17—C18—N3	118.6 (3)
S10—C6—S8	123.9 (2)	C18—C19—C14	118.5 (3)
S10—C6—S9	124.0 (2)	C18—C19—H19	120.8
S8—C6—S9	112.1 (2)	C14—C19—H19	120.8
C9—C8—C12	119.7 (3)	N1—C20—C8	178.6 (4)
			
S5—Ni1—S4—C2	−3.08 (12)	C6—S9—C5—C4	−0.8 (3)
S6—Ni1—S4—C2	177.50 (12)	C6—S9—C5—S7	−179.6 (2)
S7—Ni1—S5—C3	−177.63 (12)	C4—S8—C6—S10	−179.0 (2)
S4—Ni1—S5—C3	2.52 (12)	C4—S8—C6—S9	1.1 (2)
S7—Ni1—S6—C4	−2.01 (12)	C5—S9—C6—S10	179.7 (2)
S4—Ni1—S6—C4	177.84 (12)	C5—S9—C6—S8	−0.4 (2)
S5—Ni1—S7—C5	−176.49 (12)	C12—C8—C9—C10	0.2 (5)
S6—Ni1—S7—C5	2.94 (12)	C20—C8—C9—C10	178.5 (3)
C2—S2—C1—S1	−177.9 (2)	C11—N2—C10—C9	2.0 (5)
C2—S2—C1—S3	2.8 (2)	C13—N2—C10—C9	179.5 (3)
C3—S3—C1—S1	177.6 (2)	C8—C9—C10—N2	−1.7 (5)
C3—S3—C1—S2	−3.1 (2)	C10—N2—C11—C12	−0.7 (5)
Ni1—S4—C2—C3	3.3 (3)	C13—N2—C11—C12	−178.4 (3)
Ni1—S4—C2—S2	−175.84 (18)	N2—C11—C12—C8	−0.8 (5)
C1—S2—C2—C3	−1.3 (3)	C9—C8—C12—C11	1.0 (5)
C1—S2—C2—S4	177.9 (2)	C20—C8—C12—C11	−177.3 (3)
S4—C2—C3—S5	−1.5 (4)	C10—N2—C13—C15	−93.8 (3)
S2—C2—C3—S5	177.70 (18)	C11—N2—C13—C15	83.8 (4)
S4—C2—C3—S3	−179.97 (18)	C19—C14—C15—C16	0.7 (5)
S2—C2—C3—S3	−0.7 (4)	C19—C14—C15—C13	−176.3 (3)
Ni1—S5—C3—C2	−1.2 (3)	N2—C13—C15—C14	93.1 (3)
Ni1—S5—C3—S3	177.12 (18)	N2—C13—C15—C16	−84.0 (4)
C1—S3—C3—C2	2.4 (3)	C14—C15—C16—C17	−1.4 (5)
C1—S3—C3—S5	−176.0 (2)	C13—C15—C16—C17	175.7 (3)
Ni1—S6—C4—C5	0.3 (3)	C15—C16—C17—C18	1.1 (5)
Ni1—S6—C4—S8	−177.69 (18)	C16—C17—C18—C19	−0.1 (5)
C6—S8—C4—C5	−1.7 (3)	C16—C17—C18—N3	−179.2 (3)
C6—S8—C4—S6	176.4 (2)	O1—N3—C18—C19	−179.0 (3)
S6—C4—C5—S7	2.4 (4)	O2—N3—C18—C19	−0.1 (4)
S8—C4—C5—S7	−179.46 (18)	O1—N3—C18—C17	0.1 (4)
S6—C4—C5—S9	−176.42 (18)	O2—N3—C18—C17	179.0 (3)
S8—C4—C5—S9	1.7 (4)	C17—C18—C19—C14	−0.5 (5)
Ni1—S7—C5—C4	−3.7 (3)	N3—C18—C19—C14	178.5 (3)
Ni1—S7—C5—S9	175.08 (17)	C15—C14—C19—C18	0.2 (5)
